# EZH1/2 dual inhibitors suppress HTLV-1-infected cell proliferation and hyperimmune response in HTLV-1-associated myelopathy

**DOI:** 10.3389/fmicb.2023.1175762

**Published:** 2023-06-12

**Authors:** Akihito Koseki, Natsumi Araya, Makoto Yamagishi, Junji Yamauchi, Naoko Yagishita, Naoki Takao, Katsunori Takahashi, Yasuo Kunitomo, Daisuke Honma, Kazushi Araki, Kaoru Uchimaru, Tomoo Sato, Yoshihisa Yamano

**Affiliations:** ^1^Department of Neurology, St. Marianna University School of Medicine, Kawasaki, Japan; ^2^Department of Neurology, Yaizu City Hospital, Yaizu, Japan; ^3^Department of Rare Diseases Research, Institute of Medical Science, St. Marianna University School of Medicine, Kawasaki, Japan; ^4^Laboratory of Tumor Cell Biology, Department of Computational Biology and Medical Sciences, Graduate School of Frontier Sciences, The University of Tokyo, Tokyo, Japan; ^5^Oncology Research Laboratories, Daiichi Sankyo, Co., Ltd., Tokyo, Japan; ^6^Early Clinical Development Department, Daiichi Sankyo, Co., Ltd., Tokyo, Japan

**Keywords:** HTLV-1, HTLV-1-infected cells, HTLV-1 associated myelopathy (HAM), EZH2, epigenetic drug, valemetostat

## Abstract

**Background:**

Human T-cell leukemia virus type 1 (HTLV-1) causes HTLV-1-associated myelopathy (HAM), adult T-cell leukemia/lymphoma (ATL), HTLV-1-associated uveitis, and pulmonary diseases. Although both HAM and ATL show proliferation of infected cells, their pathogeneses are quite different. In particular, the pathogenesis of HAM is characterized by hyperimmune responses to HTLV-1-infected cells. Recently, we demonstrated the overexpression of histone methyltransferase EZH2 in ATL cells and the cytotoxic effects of EZH2 inhibitors and EZH1/2 dual inhibitors on these cells. However, these phenomena have never been studied in HAM. Furthermore, what effect these agents have on the hyperimmune response seen in HAM is completely unknown.

**Methods:**

In this study, we investigated histone methyltransferase expression levels in infected cell populations (CD4^+^ and CD4^+^CCR4^+^ cells) from patients with HAM using microarray and RT-qPCR analyses. Next, using an assay system that utilizes the spontaneous proliferation characteristic of peripheral blood mononuclear cells derived from patients with HAM (HAM-PBMCs), we investigated the effects of EZH2 selective inhibitors (GSK126 and tazemetostat) and EZH1/2 dual inhibitors (OR-S1 and valemetostat, also known as DS-3201), particularly on cell proliferation rate, cytokine production, and HTLV-1 proviral load. We also examined the effect of EZH1/2 inhibitors on the proliferation of HTLV-1-infected cell lines (HCT-4 and HCT-5) derived from patients with HAM.

**Results:**

We found elevated expression of EZH2 in CD4^+^ and CD4^+^CCR4^+^ cells from patients with HAM. EZH2 selective inhibitors and EZH1/2 inhibitors significantly inhibited spontaneous proliferation of HAM-PBMC in a concentration-dependent manner. The effect was greater with EZH1/2 inhibitors. EZH1/2 inhibitors also reduced the frequencies of Ki67^+^ CD4^+^ T cells and Ki67^+^ CD8^+^ T cells. Furthermore, they reduced HTLV-1 proviral loads and increased IL-10 levels in culture supernatants but did not alter IFN-γ and TNF-α levels. These agents also caused a concentration-dependent inhibition of the proliferation of HTLV-1-infected cell lines derived from patients with HAM and increased annexin-V(+)7-aminoactinomycin D(−) early apoptotic cells.

**Conclusion:**

This study showed that EZH1/2 inhibitors suppress HTLV-1-infected cell proliferation through apoptosis and the hyperimmune response in HAM. This indicates that EZH1/2 inhibitors may be effective in treating HAM.

## 1. Introduction

Human T-cell leukemia virus type 1 (HTLV-1) causes HTLV-1-associated myelopathy (HAM), adult T-cell leukemia/lymphoma (ATL), HTLV-1-associated uveitis, and pulmonary diseases (Poiesz et al., 1980; Hinuma et al., 1981; Gessain et al., 1985; Osame et al., 1986; Sugimoto et al., 1987; Mochizuki et al., 1992). This virus mainly infects CD4^+^ T cells, with a particularly high infection rate for CCR4^+^CD4^+^ T cells (Yoshie et al., 2002; Yamano et al., 2009; Araya et al., 2014). Although HAM and ATL are characterized by an increase in HTLV-1-infected cells, their pathogeneses differ completely. The pathogenesis of ATL involves the uncontrolled proliferation of ATL cells that HTLV-1-infected cells become through tumorigenesis. In contrast, the pathogenesis of HAM is believed to be due to an excessive immune response to HTLV-1-infected cells, resulting in the destruction and degeneration of spinal cord tissue (Yamano and Sato, 2012; Bangham et al., 2015). Indeed, in the spinal cord of patients with HAM, HTLV-1 infection has been identified only in infiltrating T cells, indicating that neurons are not infected with HTLV-1 (Moritoyo et al., 1996; Matsuoka et al., 1998; Matsuura et al., 2015). Additionally, the neuropathological analysis revealed that the spinal cord of HAM patients with short disease duration has active lesions infiltrated by both CD4^+^ T cells, including HTLV-1-infected cells, and CD8^+^ T cells. However, in patients with long disease duration, inflammatory infiltrates decreased, and CD8^+^ T cells predominated over CD4^+^ cells (Izumo et al., 1997). Based on these findings, HAM is believed not to be a neuroinfectious disease but a chronic inflammatory disease whose pathogenesis involves the formation of chronic inflammatory lesions due to a hyperimmune response to HTLV-1-infected T cells infiltrating the spinal cord (Yamano and Sato, 2012; Bangham et al., 2015). This excessive immune response is associated with high expression of the HTLV-1 tax protein, overproduction of the pro-inflammatory cytokine IFN-γ, decrease in Foxp3 expression, and suppression of regulatory T cell function in infected T cells of patients with HAM (Yamano et al., 2002, 2009; Araya et al., 2014).

Corticosteroids are currently used in the treatment of HAM to suppress this excessive immune response and have been reported to show efficacy to some extent (Coler-Reilly et al., 2017; Yamauchi et al., 2022), but they do not reduce the number of infected cells, which is the underlying cause of the hyperimmune response, nor do they sufficiently suppress the progression of symptoms (Tsutsumi et al., 2019). Given these current limitations of corticosteroid therapy, there is a strong need to develop a novel treatment for HAM that targets HTLV-1-infected cells and suppresses the excessive immune response seen in the disease.

ATL is one of the leukemias with the worst prognosis, but new drug development targeting it is quite active, which is not the case with HAM. Recently, some new drugs (such as the anti-CCR4 antibody mogamulizumab, immunomodulator lenalidomide, histone deacetylase inhibitor tucidinostat, and histone methyltransferase EZH1/2 dual inhibitor valemetostat) have become available in Japan for the treatment of ATL (Ishida et al., 2012, 2016; Izutsu et al., 2022; Utsunomiya et al., 2022). Enhancer of zeste homolog 2 (EZH2) and its close homolog EZH1, the targets of valemetostat, are enzymatic subunits of the polycomb repressive complex 2 that is responsible for trimethylation of lysine 27 of histone H3 (H3K27me3) (Margueron and Reinberg, 2011). We have previously shown that EZH2 overexpression and the resulting H3K27me3 alteration occur not only in ATL cells but also in tax-expressing cells (Fujikawa et al., 2016) and that EZH1/2 dual inhibitors are more effective than EZH2 selective inhibitors in depleting HTLV-1-infected cell populations (Yamagishi et al., 2019). However, there have been no reports that EZH2 or EZH1/2 inhibitors are considered potential therapeutic agents for HAM. Therefore, it is still unclear how these agents act on HTLV-1-infected cells derived from patients with HAM and how they affect the excessive immune response seen in HAM. Thus, this study aimed to investigate whether EZH2 and EZH1/2 inhibitors can inhibit the proliferation of HTLV-1-infected cells derived from patients with HAM and the excessive immune response seen in HAM *in vitro* and to examine their potential as novel therapeutic agents for HAM. To achieve this objective, we investigated three parameters in this study: (1) quantitative expression of EZH2 and EZH1 in cell populations containing HTLV-1-infected cells from patients with HAM; (2) effects of EZH2 and EZH1/2 inhibitors on spontaneous proliferation of peripheral blood mononuclear cells (PBMCs) from patients with HAM, used as an *in vitro* model of excessive immune response in HAM, particularly on cell proliferation rate, cytokine production, and HTLV-1 proviral load; and (3) changes in viability and apoptotic cell rate of HTLV-1-infected cell lines derived from patients with HAM due to EZH1/2 inhibitors.

## 2. Materials and methods

### 2.1. Subjects

The study used 9 blood samples from 8 healthy donors (HDs; 4 men and 4 women; mean age, 39 years) and 25 blood samples from 21 patients with HAM (5 men and 16 women; mean age, 69 years). Patients with HAM were diagnosed according to World Health Organization guidelines (Osame, 1990). PBMCs were separated using Pancoll^®^ density gradient centrifugation (density: 1.077 g/mL; PANBiotech GmbH, Aidenbach, Germany). The separated PBMCs were frozen in cryopreserving fluid (Cell Banker 1; Mitsubishi Chemical Medience Corporation, Tokyo, Japan) and stored in liquid nitrogen. This study was approved by the Bioethics Committee of the St. Marianna University School of Medicine (Approval ID No. 1646). All participants gave their written informed consent.

### 2.2. Microarray analysis

PBMCs from HDs (*n* = 4) and patients with HAM (*n* = 4) were used as starting materials. CD4^+^ T cells were negatively isolated from the PBMCs using a human CD4^+^ T cell isolation kit (Miltenyi Biotec, Bergisch Gladbach, Germany). After removing the MACS column from the separator, the cells were flushed out and used as CD4^+^ T cell-depleted PBMCs. Subsequent manipulations were performed using a previously reported method (Araya et al., 2014). Briefly, total RNA was prepared using TRIZOL (Invitrogen, Carlsbad, CA, United States). Each RNA was amplified and labeled with cyanine 3, using the Agilent Quick Amp Labeling Kit, 1-color (Agilent Technologies, Santa Clara, CA, United States). Cyanine 3-labeled cRNA was fragmented and hybridized to an Agilent Human GE 4x44K Microarray (design ID 014850) loaded with a total of 41,000 probes, excluding control probes. After washing, the microarray was scanned using an Agilent DNA microarray scanner. Intensity values for each feature scanned were quantified using Agilent feature extraction software (version 9.5.3.1) with background subtraction. All data were analyzed using GeneSpring GX software (Agilent Technologies). The box plots were analyzed and visualized using R version 4.2.2. Clustering was performed using R package gplots (distance, Pearson correlation; linkage rule, Ward’s method). Gene Ontology analysis was performed by DAVID Bioinformatics Resources.^1^

### 2.3. Reverse transcriptase-quantitative PCR

CD4^+^CCR4^+^ cells were isolated from the PBMCs of HDs (*n* = 5) and patients with HAM (*n* = 5) using a previously described method (Araya et al., 2014). Total RNA was extracted from isolated CD4^+^CCR4^+^ cells, and cDNA was generated using ReverTra Ace (TOYOBO, Osaka, Japan). Using the prepared cDNA as a template, the expression levels of EZH2 in CD4^+^CCR4^+^ cells of HDs and patients with HAM were analyzed via quantitative PCR. Primer sets and a probe used to detect EZH2 expression were EZH2#35-F (TGTGGATACTCCTCCAAGGAA), EZH2#35-R (GAGGAGCCGTCCTTTTTCA), and Universal ProbeLibrary #35 (Roche Diagnostics, Rotkreuz, Switzerland). Relative quantification of mRNA was determined with the comparative Ct method using GAPDH as an internal control (Yamano et al., 2009). The following equation was used to determine the relative expression level of the target gene: target gene expression = 2-(Ct[target] - Ct[GAPDH]).

### 2.4. Reagents

The selective EZH2 inhibitors (GSK126 and tazemetostat) and EZH1/2 dual inhibitors (OR-S1 and valemetostat) were synthesized and provided by Daiichi Sankyo, Co., Ltd. (Tokyo, Japan). Prednisolone, which was used as a positive control, was purchased from LKT Laboratories, Inc. (St. Paul, MN, United States). Dimethylsulfoxide (DMSO) was purchased from Sigma-Aldrich (St. Louis, MO, United States).

### 2.5. Cell culture

For cell proliferation assay, human PBMCs in RPMI 1640 (FUJIFILM Wako Chemicals, Osaka, Japan) supplemented with 10% heat-inactivated fetal bovine serum and 1% penicillin and streptomycin antibiotic solution (FUJIFILM) were cultured at 37°C in 5% CO_2_. For the cell viability assay, we used HTLV-1-infected cells lines (HCT-4 and HCT-5) established from cerebrospinal fluid cells of patients with HAM. HCT-4 cells were cultured in RPMI 1640 supplemented with 10% heat-inactivated fetal bovine serum, 1% L-glutamine (FUJIFILM), 1% penicillin and streptomycin antibiotic solution, and 100 U/mL IL-2 (Cell Science & Technology Institute, Inc., Sendai, Japan). HCT-5 cells were cultured in RPMI 1640 supplemented with 20% heat-inactivated fetal bovine serum, 1% L-glutamine, 1% penicillin and streptomycin antibiotic solution, and 200 U/mL IL-2.

To examine the effect of EZH1/2 inhibitors on the viability of HCT-4 and HCT-5 cells over time, the cells were cultured under the following conditions: 3 × 10^6^ HCT-4 or 2.5 × 10^6^ HCT-5 cells were seeded and cultured in 20 mL of culture medium with DMSO, 1 μM OR-S1, or 1 μM valemetostat for 21 days. Cell passages were performed as follows. First, the volume of culture medium containing 3 × 10^6^ HCT-4 or 2.5 × 10^6^ HCT-5 cells in the DMSO-treated groups was calculated. Next, in all groups, the calculated volume of the cell culture medium was transferred to a new flask, and the fresh medium was added up to 20 mL. Finally, DMSO, OR-S1, or valemetostat was added again. Cells were collected from the culture medium 7, 11, 14, and 21 days after the initiation of the culture and used for cell viability assay.

To examine the effect of various concentrations of EZH1/2 inhibitors on the viability of HCT-4 and HCT-5 cells, cell culture was also performed as follows: 4.5 × 10^5^ HCT-4 or 3.75 × 10^5^ HCT-5 cells were seeded in 6-well plates and cultured in 3 mL of culture medium with DMSO, OR-S1 dilution series, or valemetostat dilution series for 14 days, with repeated passages every 3 to 4 days. The passages were performed in the same manner as described above, except for the number of cells. The number of seeded cells was 4.5 × 10^5^ cells for HCT-4 and 3.75 × 10^5^ cells for HCT-5. Cells were collected from the culture medium 14 days after culture initiation for the cell viability assay.

### 2.6. Cell proliferation assay

PBMCs from patients with HAM (*n* = 8) were seeded at 1 × 10^5^ cells/well in 96-well round bottom plates. They were cultured for 7 days in the presence or absence of OR-S1, valemetostat, GSK126, tazemetostat, or prednisolone. During the last 16 h, 1 μCi of ^3^H-thymidine was added to each well, and then the cells were harvested and counted using a 2450 MicroBeta^2^ Plate Counter (Perkin Elmer, Boston, MA). The assay was performed in triplicate. To verify the effect of the EZH1/2 inhibitors, PBMCs from another eight patients with HAM were also used. Using the average counts of ^3^H-thymidine incorporation in the DMSO-treated group as 100%, the average of the relative values in each drug-treated group was calculated and defined as the ^3^H-thymidine incorporation rate (%). The IC50 of each drug was calculated by substituting the data obtained from this experiment into the following equation: IC50 = 10ˆ[LOG(A/B)∗(50-C)/(D-C) + LOG(B)], where A is the higher concentration considering the two values that sandwich 50% of ^3^H-thymidine incorporation rate, B is the lower concentration considering the same two values, C is the ^3^H-thymidine incorporation rate (%) determined for B, and D is the ^3^H-thymidine incorporation rate (%) determined for A.

### 2.7. Measurement of cytokines and HTLV-1 proviral load

PBMCs from patients with HAM (*n* = 8) were seeded at 5 × 10^5^ cells/well in 48-well plates. They were cultured for 12 days in the presence or absence of OR-S1, valemetostat, or prednisolone. Supernatants were collected and stored at −80°C. The cells were then harvested for DNA extraction or flow cytometric analysis. The concentrations of IFN-γ, TNF-α, IL-6, and IL-10 in the culture supernatants were measured with a cytometric bead array kit (BD Biosciences, Franklin Lakes, NJ, United States), using a flow cytometer FACSCantoII (BD Biosciences) according to the manufacturer’s instructions. HTLV-1 proviral loads were measured with ABI Prism 7500 SDS (Applied Biosystems, Carlsbad, CA, United States) using a previously described method (Yamano et al., 2002). HTLV-1 (pX) per 100 cells = (copy number of pX) / ((copy number of β-actin)/2) × 100.

### 2.8. Flow cytometric analysis

Zombie NIR™ Fixable Viability Kit (BioLegend, San Diego, CA, United States) was used to exclude dead cells. PBMCs were immunostained with a combination of the following fluorescence-conjugated antibodies to cell surface markers: CD3 (UCHT1), CD4 (OKT4), and CD8 (RPA-T8). Cells were fixed with 70% EtOH for 90 min at −20°C, then intracellularly stained with Ki67 antibodies (MOPC-21). The stained cells were analyzed using FACSCantoII (BD Biosciences). Data were processed using FlowJo software (BD biosciences).

### 2.9. Cell viability assay

HCT-4 and HCT-5 cells were cultured as described in Section 2.5. To measure cell viability, 100 μL of cell suspensions were seeded in 96-well plates, 10 μL of cell counting kit-8 (CCK-8) (Dojindo Laboratories, Kumamoto, Japan) was added to each well, and after 3 h of incubation at 37°C, the absorbance at 450 nm was measured using an iMark microplate reader (Bio-Rad Laboratories, Hercules, CA, United States). Cell viability was calculated using the following formula: cell viability = (experimental optical density value - blank optical density value) / (DMSO-treated control optical density value - blank optical density value) × 100%. The IC50 of each drug in the cell viability assay was calculated using the same method as for the cell proliferation assay.

### 2.10. Apoptotic cell analysis

HCT-4 or HCT-5 cells were cultured for 21 days with 1 μM OR-S1 or 1 μM valemetostat. The harvested cells were stained with PE annexin V and 7-aminoactinomycin D (7-AAD) according to the instructions provided with PE Annexin V Apoptosis Detection Kit I (BD Biosciences) and analyzed with a BD FACSCantoII (BD Biosciences). The flow cytometric data was processed using FlowJo software (BD Biosciences).

### 2.11. Statistical analysis

The unpaired t-test was used to compare *EZH2* mRNA expression in CD4^+^CCR4^+^ cells between HDs and patients with HAM. The Friedman test for repeated measurements was used, followed by the Dunn multiple comparison test, to analyze differences among various drug concentrations. Statistical analysis was performed using GraphPad Prism 7 (GraphPad Software, San Diego, CA, United States). Statistical significance was set at a *p*-value <0.05.

## 3. Results

### 3.1. Upregulation of *EZH2* gene expression in CD4^+^ T cells of patients with HAM

**Figure 1:**
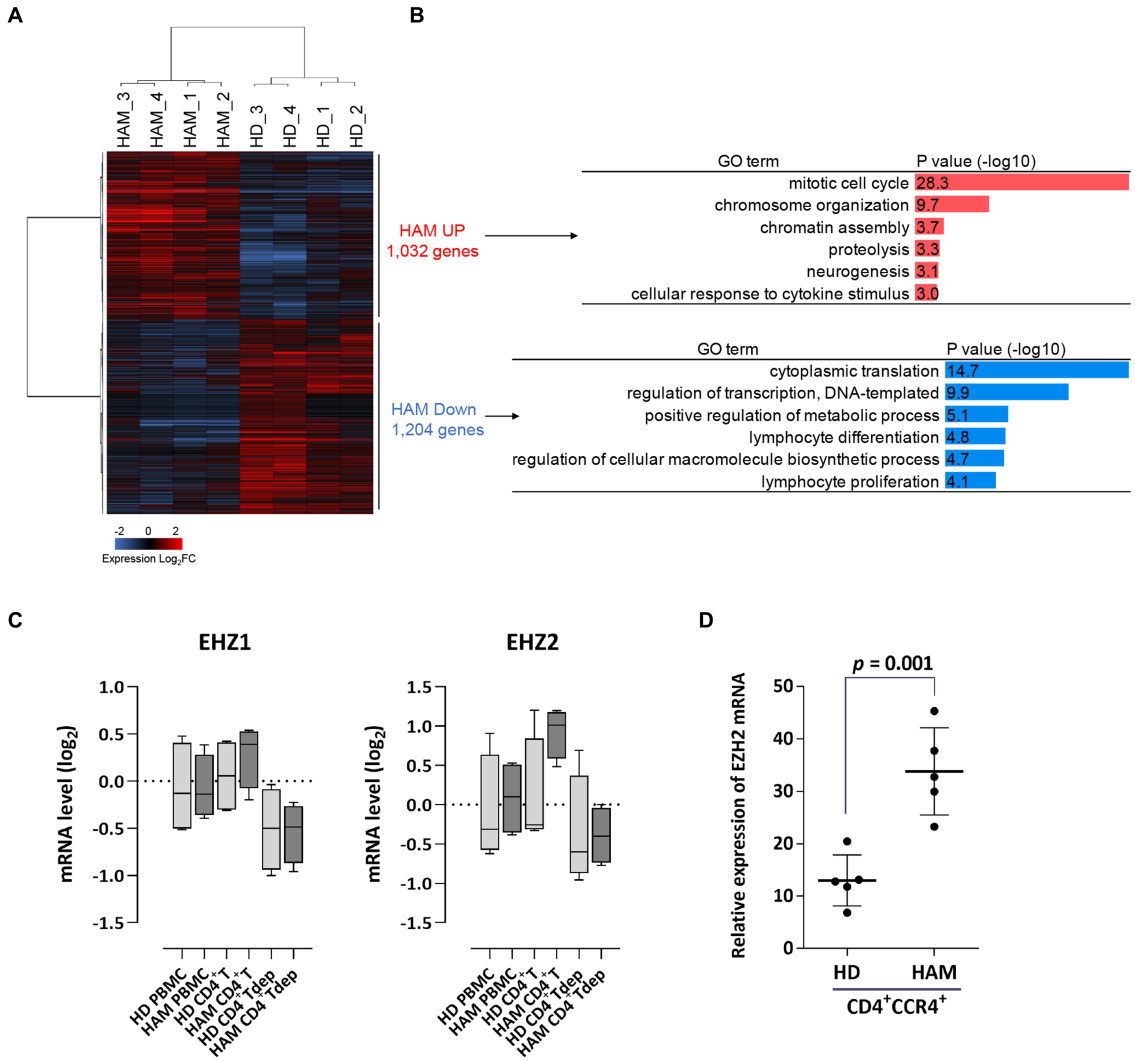
Upregulation of *EZH2* gene expression in CD4^+^ T cells of patients with HAM. **(A)** Differential expression analysis of CD4^+^ T cells in HAM patients (HAM, *n* = 4) and healthy donors (HD, *n* = 4). A heat map and hierarchical clustering of 2,236 differentially expressed genes (1,032 upregulated and 1,204 downregulated) is shown (log_2_ fold change [FC]). **(B)** Gene ontology (GO) analysis of the upregulated and downregulated genes in HAM CD4^+^ T cells. Bar charts show the top 6 GO terms for biological processes. **(C)** *EZH1* and *EZH2* mRNA expression levels in microarray data obtained from peripheral blood mononuclear cells (PBMCs), CD4^+^ T cells, and CD4^+^ T cell-depleted PBMCs (CD4^+^Tdep) from healthy donors (HDs) (*n* = 4) and patients with HTLV-1-associated myelopathy (HAM) (*n* = 4). The y-axis indicates log2 transformed mRNA expression level. Data are presented as box and whisker plots showing the median, lower and upper quartiles, and the minimum-maximum. **(D)** The dot plot (mean ± standard deviation) indicates *EZH2* mRNA expression levels in CD4^+^CCR4^+^ T cells from HDs (*n* = 5) and patients with HAM (*n* = 5), as measured via RT-qPCR. Data were analyzed using an unpaired t-test.

Microarray analysis revealed common gene expression patterns in CD4^+^ T cells from patients with HAM (Figure 1A). The gene ontology analysis of differentially expressed genes showed that most upregulated genes belonged to the mitotic cell cycle, which is closely related to cell proliferation. In contrast, downregulated genes were involved not only in cytoplasmic translation but also in transcriptional regulation and T-cell differentiation (Figure 1B). Based on the microarray data, the median expression levels of the *EZH1* and *EZH2* genes in CD4^+^ T cells from patients with HAM were 1.3- and 2.4-fold higher, respectively, than in CD4^+^ T cells from HDs (Figure 1C). Next, *EZH2* mRNA expression levels in CD4^+^CCR4^+^ T cells from other patients with HAM (n = 5) were examined using RT-qPCR and found to be 2.6 times higher than to those from HDs (*p* = 0.001, Figure 1D). Furthermore, we examined our previous microarray data (Accession No. GSE57259) and found that *EZH1* and *EZH2* in CD4^+^CD25^+^CCR4^+^ T cells from patients with HAM were 0.94- and 3.9-fold higher than those in HDs, respectively.

### 3.2. Inhibitory effects of EZH2 inhibitors and EZH1/2 inhibitors on spontaneous proliferation of PBMCs from patients with HAM

**Figure 2:**
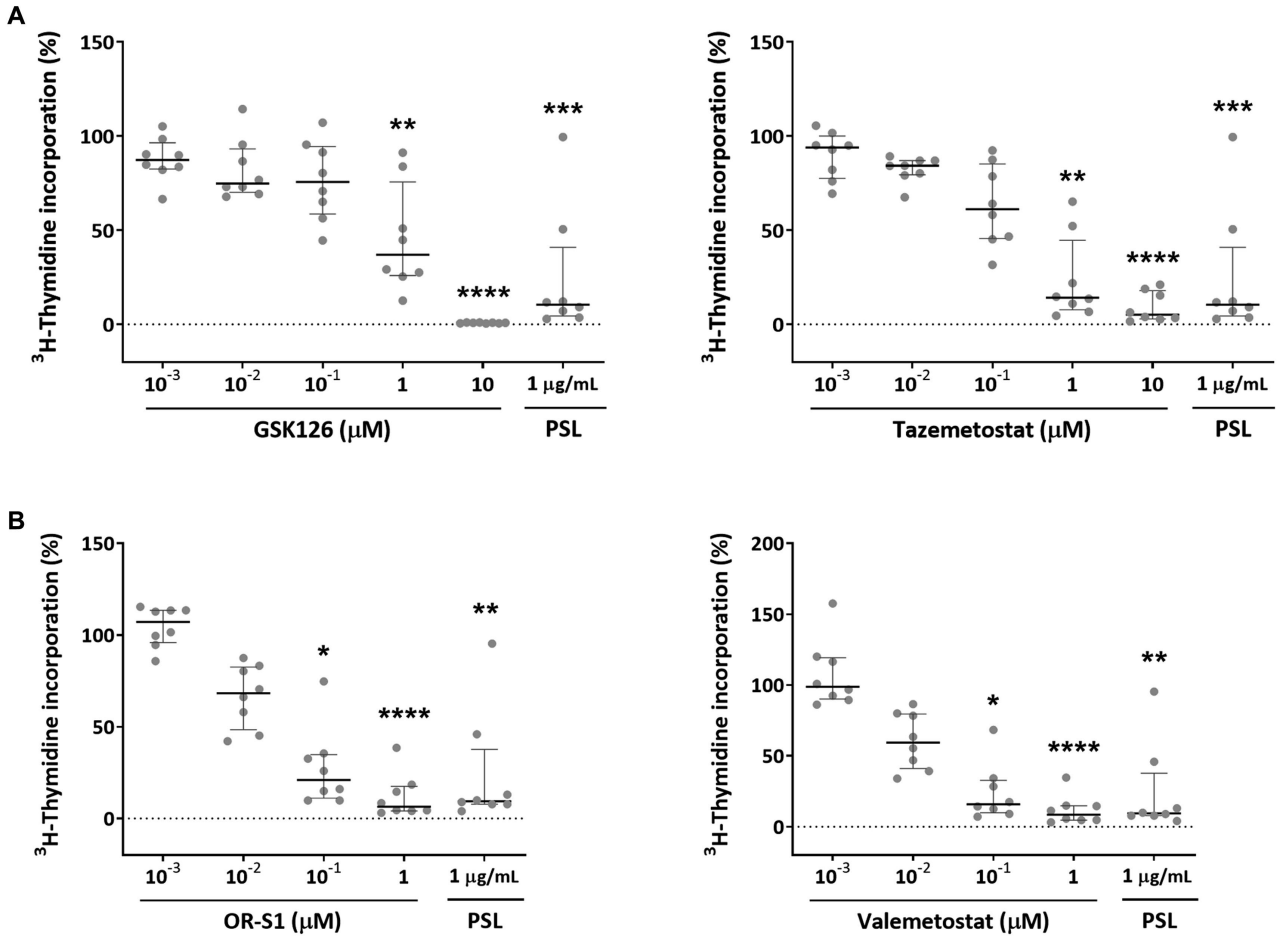
Inhibitory effects of EZH2 inhibitors and EZH1/2 dual inhibitors on spontaneous proliferation of PBMCs from patients with HAM. To examine the effect of **(A)** EZH2 selective inhibitors (GSK126 and tazemetostat) and **(B)** EZH1/2 dual inhibitors (OR-S1 and valemetostat) on the excessive immune response that is characteristic of HTLV-1-associated myelopathy (HAM), ^3^H-thymidine incorporation into DNA in peripheral blood mononuclear cells (PBMCs) from patients with HAM (*n* = 8), in the presence of each inhibitor, was measured after 7 days of culture. The rate of ^3^H-thymidine incorporation indicates relative values with the value of the DMSO-treated group set as 100%. Thick horizontal lines and error bars represent the median and interquartile range. Prednisolone (PSL) at 1 μg/mL was used as a positive control. Statistical analysis was performed using the Friedman test, followed by the Dunn test for multiple comparisons. ^*^ < 0.05, ^**^ < 0.01, ^***^ < 0.001, ^****^ < 0.0001.

We evaluated the effects of EZH2 selective inhibitors and EZH1/2 dual inhibitors on the spontaneous proliferation of HAM patients’ PBMCs that could proliferate in the absence of mitogens or exogenous growth factors. Since this phenomenon is known to be inhibited by corticosteroids (Itoyama et al., 1988; Ijichi et al., 1989), prednisolone was used as a positive control. Experimental results demonstrated that EZH2 inhibitors (GSK126 and tazemetostat) inhibited spontaneous PBMC proliferation in patients with HAM (*n* = 8) in a concentration-dependent manner (Figure 2A). Both drugs significantly inhibited it at 1 μM (*p* < 0.01 for both). The IC50 values of these drugs were 724.3 nM and 214.2 nM, respectively. EZH1/2 dual inhibitors (OR-S1 and valemetostat) also showed a concentration-dependent inhibition of spontaneous proliferation of PBMCs from the same patients with HAM (Figure 2B). Both drugs significantly inhibited it at 0.1 μM (*p* < 0.05 for both).

Their IC50 values were 26.5 nM and 19.4 nM, respectively. The inhibitory effect of OR-S1 and valemetostat on the spontaneous proliferation of PBMCs from other patients with HAM (n = 8) was also observed, confirming the reproducibility of the inhibitory effect, and both drugs showed significant inhibition at 0.1 μM (p < 0.05 and *p* < 0.01, respectively) (Supplementary Figure S1).

### 3.3. Proliferation inhibition of CD4^+^ T cells and CD8^+^ T cells by EZH1/2 inhibitors during spontaneous proliferation of PBMCs from patients with HAM

**Figure 3:**
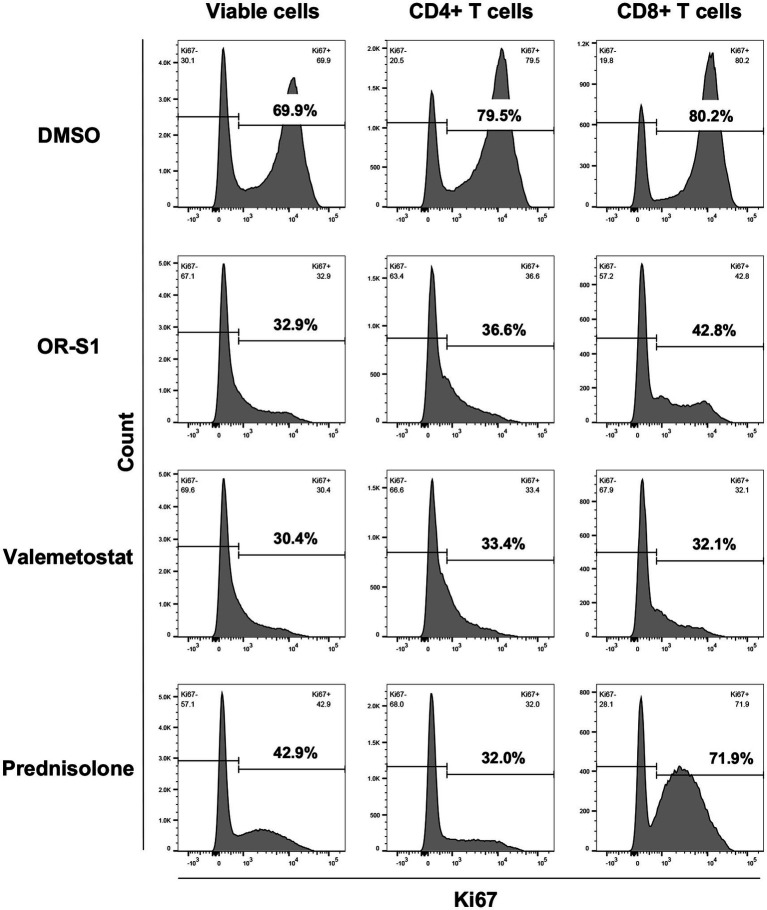
Proliferation inhibition of CD4^+^ T cells and CD8^+^ T cells by EZH1/2 inhibitors during spontaneous proliferation of PBMCs from patients with HAM. Peripheral blood mononuclear cells (PBMCs) from a patient with HTLV-1-associated myelopathy (HAM) were cultured for 7 days in the presence of dimethyl sulfoxide (DMSO, 1:10000 dilution), OR-S1 (1 μM), valemetostat (1 μM), or prednisolone (1 μg/mL). The rate of Ki67 expression, a marker for cell proliferation, is shown for all viable cells (left column), CD4^+^ T cells (middle column), and CD8^+^ T cells (right column) from the cultured PBMCs of a representative HAM patient.

EZH1/2 inhibitors and prednisolone reduced the percentage of Ki67^+^ cells in all viable cells on day 7 after culture (Figure 3 and Supplementary Figure S2). Further analysis revealed that all drugs could reduce the percentage of Ki67^+^ cells in CD4^+^ T cells and in CD8^+^ T cells. The pattern of proliferation inhibition of CD4^+^ T cells and CD8^+^ T cells by EZH1/2 inhibitors was different from that by prednisolone. Valemetostat had a higher inhibitory effect than OR-S1 in all three patients (Figure 3 and Supplementary Figure S2).

### 3.4. Increase in IL-10 production by EZH1/2 inhibitors during spontaneous proliferation of PBMCs from patients with HAM

**Figure 4:**
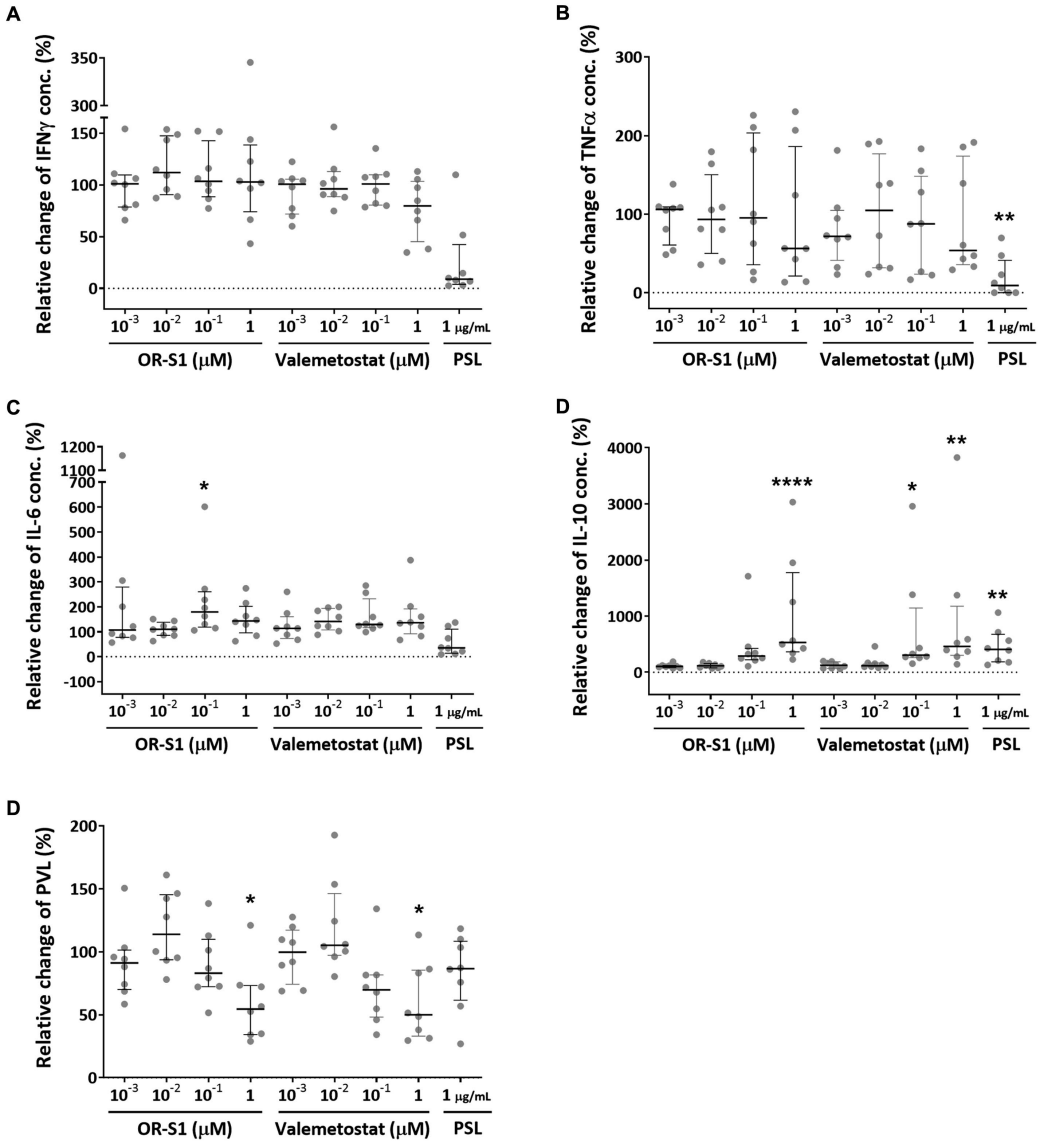
Impact of EZH1/2 dual inhibitors on cytokine production and HTLV-1 proviral load on the spontaneous proliferation of PBMCs from patients with HAM. Peripheral blood mononuclear cells (PBMCs) from patients with HTLV-1-associated myelopathy (HAM) (*n* = 8) were cultured for 12 days in the presence of OR-S1, valemetostat, or prednisolone (PSL). The concentrations of IFN-γ **(A)**, TNF-α **(B)**, IL-6 **(C)**, and IL-10 **(D)** in the culture supernatants were determined with the CBA method. The HTLV-1 proviral load (PVL) **(E)** in the cells was also measured using real-time PCR. Data indicate relative values with the value of the DMSO-treated group set as 100%. Thick horizontal lines and error bars represent the median and interquartile range. Statistical analysis was performed using the Friedman test, followed by the Dunn test for multiple comparisons. ^*^ < 0.05, ^**^ < 0.01, ^***^ < 0.001, ^****^ < 0.0001.

To evaluate the effect of OR-S1 and valemetostat on cytokine production, we measured the concentrations of the pro-inflammatory cytokines IFN-γ, TNF-α, and IL-6 and the anti-inflammatory cytokine IL-10 in the culture supernatants of PBMCs of patients with HAM (n = 8) on day 12 after culture, in the presence or absence of each drug. Prednisolone at 1 μg/mL, the positive control, showed a tendency to decrease IFN-γ and IL-6 levels, and it significantly decreased TNF-α level (*p* < 0.01) (Figures 4A–C). OR-S1 and valemetostat hardly changed IFN-γ and TNF-α production. There was a wide variation in the change in IL-6 production by OR-S1, but only 0.1 μM OR-S1 produced a significant change (*p* < 0.05). OR-S1 and valemetostat increased IL-10 levels in a concentration-dependent manner (Figure 4D). The increase was significant in response to 1 μg/mL prednisolone, 1 μM OR-S1, 100 nM valemetostat, and 1 μM valemetostat (p < 0.01, *p* < 0.0001, *p* < 0.05, and *p* < 0.01, respectively).

### 3.5. Decrease in HTLV-1 proviral load affected by EZH1/2 inhibitors during spontaneous proliferation of PBMCs from patients with HAM

To evaluate the effect of OR-S1 and valemetostat on HTLV-1 proviral load, we measured the HTLV-1 proviral loads in PBMCs of patients with HAM (*n* = 8) on day 12 after culture, in the presence or absence of each drug. Prednisolone did not significantly reduce proviral loads, as previously reported (Figure 4E). OR-S1 and valemetostat showed a tendency to decrease HTLV-1 proviral load at 0.1 μM and significantly decreased it at 1 μM.

### 3.6. Time-and dose-dependent reduction of viability of HTLV-1-infected cell lines derived from patients with HAM by EZH1/2 inhibitors

**Figure 5:**
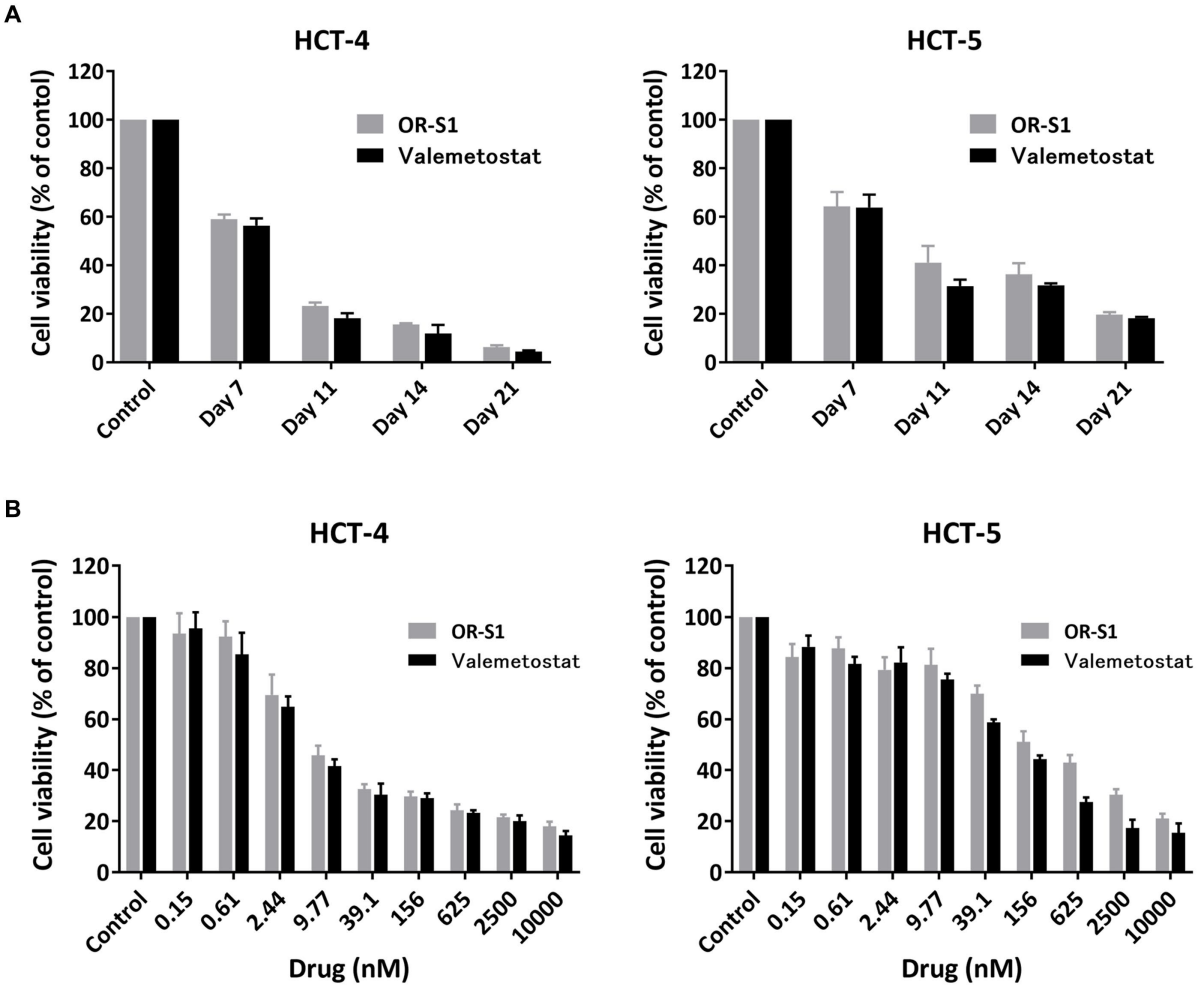
Time-and dose-dependent reduction of viability of HTLV-1-infected cell lines derived from patients with HAM by EZH1/2 inhibitors. **(A)** Viability of HTLV-1-infected cells lines (HCT-4 and HCT-5) derived from patients with HTLV-1-associated myelopathy (HAM) cultured in the presence of 1 μM OR-S1 or valemetostat on days 7, 11, 14, and 21. **(B)** The viability of HCT-4 and HCT-5 cells cultured for 14 days in the presence of nine concentrations of OR-S1 or valemetostat, ranging from 10,000 nM to 0.15 nM. Cell viability was calculated based on the absorbance of water-soluble tetrazolium 8, with the DMSO-treated group used as a control in each condition. Data are shown as mean ± standard deviation.

To investigate the potential of OR-S1 and valemetostat to kill HTLV-1-infected cells, we used HCT-4 and HCT-5 cells that were HTLV-1-infected cell lines established from cerebrospinal fluid cells from patients with HAM. The viability of HCT-4 and HCT-5 cells decreased over time in the presence of 1 μM OR-S1 or 1 μM valemetostat (Figure 5A) and also decreased in a concentration-dependent manner with OR-S1 or valemetostat (Figure 5B). The IC50 values of OR-S1 and valemetostat for HCT-4 cells were 7.63 nM and 5.92 nM, respectively, and those of HCT-5 cells were 185.4 nM and 90.6 nM, respectively.

### 3.7. Apoptosis induction by EZH1/2 inhibitors in HTLV-1-infected cell lines derived from patients with HAM

**Figure 6:**
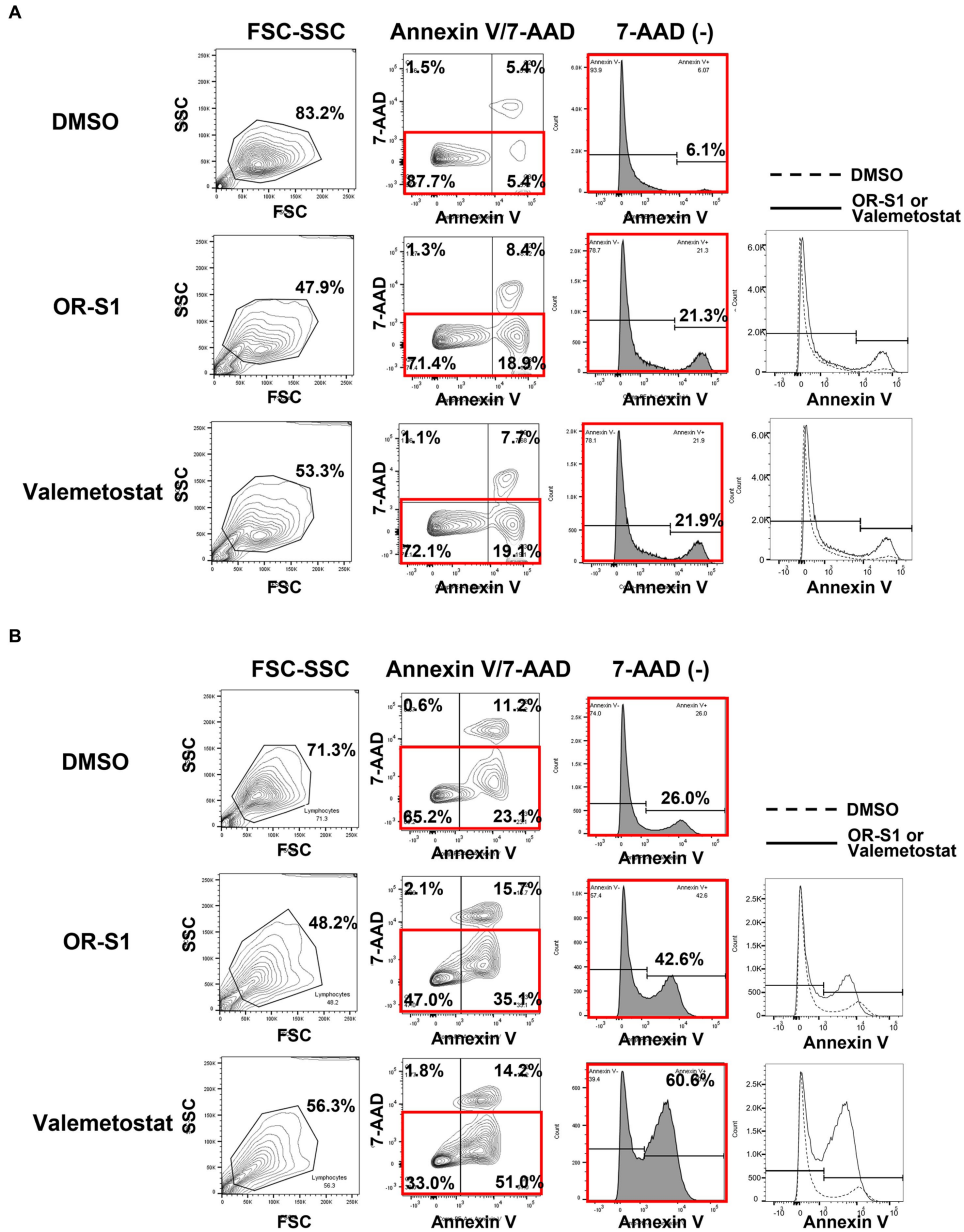
Apoptosis induction by EZH1/2 inhibitors in HTLV-1-infected cell lines derived from patients with HAM HCT-4 cells. **(A)** and HCT-5 cells **(B)** were cultured in the presence of 1 μM OR-S1 or valemetostat for 21 days. These cells were stained with PE annexin V and 7-aminoactinomycin D (7-AAD), followed by apoptotic cell analysis using a flow cytometer. DMSO: dimethylsulfoxide, FSC: forward scatter, SSC: side scatter.

To investigate the mechanism by which OR-S1 and valemetostat kill HTLV-1-infected cells, we performed apoptotic cell analysis using annexin V/7-AAD staining. The percentage of annexin V(+)7-AAD(−) HCT-4 cells were 6.1, 21.3, and 21.9% in the DMSO-, OR-S1-and valemetostat-treated groups, respectively (Figure 6A). Similarly, the percentages of annexin V(+)7-AAD(−) HCT-5 cells were 26.0, 42.6, and 60.6%, respectively (Figure 6B). Thus, an increase in early apoptotic cells was observed with EZH1/2 inhibitors.

## 4. Discussion

This *in vitro* study demonstrated that EZH1/2 dual inhibitors have cytotoxic activity on HTLV-1-infected cells derived from patients with HAM and are effective in suppressing excessive immune responses, suggesting their efficacy as therapeutic agents for HAM. Here, we will discuss the three parameters described at the end of the Introduction section. First, the microarray analysis and RT-qPCR data revealed increased expression of EZH2 in cell populations (CD4^+^T, CD4^+^CCR4^+^, and CD4^+^CD25^+^CCR4^+^ cells) containing HTLV-1-infected cells from patients with HAM. This finding is consistent with our previous report of increased EZH2 expression in cell populations (CD4^+^CADM1^+^ cells) containing infected cells from HTLV-1 carriers and patients with indolent ATL (Fujikawa et al., 2016). We speculate that one of the mechanisms for this increased expression of EZH2 is that the infected cells of patients with HAM express *tax* mRNA (Yamano et al., 2002), and tax induces the expression of EZH2 (Fujikawa et al., 2016).

Next, we found by ^3^H-thymidine incorporation assay that EZH2 and EZH1/2 inhibitors suppressed the spontaneous proliferation of HAM patient-derived PBMCs in a concentration-dependent manner. According to previous reports, this phenomenon of spontaneous PBMC proliferation in patients with HAM is thought to mimic the hyperimmune response observed in HAM, which consists of an increase in and activation of infected cells, followed by a proliferative response of CD8^+^ T cells that are specific for viral antigens, such as tax and env, which are expressed by infected cells (Itoyama et al., 1988; Ijichi et al., 1989). Just as prednisolone, which is used as a treatment for HAM, suppresses this phenomenon, the EZH2 and EZH1/2 inhibitors used in this study also inhibited it. Therefore, these drugs are likely to suppress the excessive immune response in HAM.

However, why do EZH2 and EZH1/2 inhibitors suppress the spontaneous proliferation of PBMCs in patients with HAM? There are three possible reasons: (1) these drugs reduce the number of infected cells; (2) they suppress the proliferative response of HTLV-1-specific CD8^+^ T cells; and (3) they enhance the production of IL-10, an anti-inflammatory cytokine. First, reduction in the number of infected cells is suggested by the present findings that EZH2 and EZH1/2 inhibitors reduced proviral load in PBMCs from patients with HAM (Figure 4E) and decreased the viability of HTLV-1-infected cells lines (Figure 5). It is supported by our previous findings that anti-CCR4 antibodies suppress spontaneous proliferation by killing infected cells (CD4^+^CCR4^+^ T cells) (Yamauchi et al., 2015). Second, the suppression of the proliferative response of HTLV-1-specific CD8^+^ T cells was demonstrated by flow cytometric analysis in this study (Figure 3 and Supplementary Figure S2). This is also supported by previous reports stating that EZH2 inhibition causes effector CD8^+^ T cells to undergo cell death and reduces proliferative responses (Wang et al., 2016; Stairiker et al., 2020). Third, regarding the enhanced IL-10 production by EZH1/2 inhibitors (Figure 4D), the suppressive function of IL-10 on Th1 responses may have inhibited spontaneous proliferation. However, the production of Th1 cytokines (IFN-γ and TNF-α) was not suppressed (Figures 4A,B). Thus, this mechanism requires further investigation.

Finally, we investigated the changes in viability of HTLV-1-infected cell lines derived from HAM patients due to the action of EZH1/2 inhibitors. The results showed that EZH1/2 inhibitors induced apoptosis in HTLV-1-infected cell lines and reduced their viability. This is supported by the finding that EZH1/2 dual inhibition strongly induces apoptosis by preventing cell cycle progression in the HTLV-1-infected cell line TL-Om1 (Yamagishi et al., 2019). Taken together with the fact that EZH1/2 inhibitors reduced proviral load after culture of PBMCs from patients with HAM (Figure 4E), these drugs are thought to have cytotoxic activity against HTLV-1-infected cells from patients with HAM.

There is one limitation to this study. Only EZH2 expression, and not EZH1 expression, was upregulated in cell populations containing infected cells in patients with HAM. Nevertheless, we used EZH1/2 dual inhibitors in most experiments, and EZH2 selective inhibitors were used only in the experiments to confirm their effects on spontaneous proliferation. The inhibitory effect of EZH1/2 dual inhibitors on spontaneous proliferation was stronger than that of EZH2 selective inhibitors (Figure 2). Previous reports have shown that inhibition of EZH2 alone results in residual H3K27me3 due to the compensatory action of EZH1, while inhibition of both EZH1 and EZH2 efficiently diminishes residual H3K27me3 (Yamagishi et al., 2019). Therefore, we speculate that EZH1/2 dual inhibitors are also more effective than EZH2 selective inhibitors for the other effects not tested in the present study.

In conclusion, we found that EZH1/2 inhibitors suppress HTLV-1-infected cell proliferation in patients with HAM and the excessive immune response in HAM. This indicates that they may be effective as therapeutic agents for HAM. To confirm this, proof-of-concept clinical trials should be conducted in patients with HAM in the future.

## Data availability statement

The datasets presented in this study can be found in online repositories. The names of the repository/repositories and accession number(s) can be found at: https://www.ncbi.nlm.nih.gov/geo/query/acc.cgi?acc=GSE233437.

## Ethics statement

The studies involving human participants were reviewed and approved by Bioethics Committee of St. Marianna University School of Medicine. The patients/participants provided their written informed consent to participate in this study.

## Author contributions

AK, NA, MY, KU, and YY contributed to the conception and design of the study. NA performed most of the experiments and data analysis. MY contributed to microarray analysis and its data analysis. AK, NA, and TS created the figures and wrote the manuscript. MY, JY, NY, NT, KA, KU, and YY reviewed and corrected the manuscript. All authors contributed to the article and approved the submitted version.

## Funding

This work was supported by grants from the Practical Research Project for Rare/Intractable Diseases of the Japan Agency for Medical Research and Development (AMED, Nos. JP22ek0109529, JP22ek0109441, JP22ek0109493, JP22ek0109548, JP22fk0108126, JP22wm0325017, JP22ck0106703, JP23fk0108672, and JP23wm0325056), Rare and Intractable Diseases from the Ministry of Health, Labour and Welfare of Japan (No. JPMH22FC1013), and Japan Society for the Promotion of Science (JSPS) KAKENHI (Nos. JP16H05323, JP19H03575, JP22H02987, JP22K07507, JP22K08386, and JP23K06954).

## Conflict of interest

DH and KA are employees of Daiichi Sankyo Co., Ltd. YY, MY, and KU received research funding from Daiichi Sankyo Co., Ltd. Daiichi Sankyo Co., Ltd., holds substance patents on the EZH1/2 inhibitors. St. Marianna University and Daiichi Sankyo Co., Ltd., hold patents for the application of the EZH1/2 inhibitors to HAM patients. YY and KA are named as inventors.

The remaining authors declare that the research was conducted in the absence of any commercial or financial relationships that could be construed as a potential conflict of interest.

## Publisher’s note

All claims expressed in this article are solely those of the authors and do not necessarily represent those of their affiliated organizations, or those of the publisher, the editors and the reviewers. Any product that may be evaluated in this article, or claim that may be made by its manufacturer, is not guaranteed or endorsed by the publisher.

## Footnotes

^1^ https://david.ncifcrf.gov/

## Abbreviations

7-AAD: 7-aminoactinomycin D
DMSO: dimethylsulfoxide
EZH2: enhancer of zeste homolog 2
H3K27me3: trimethylated lysine 27 of histone H3
HD: healthy donor
PBMC: peripheral blood mononuclear cell
