# Supplementary figures and images for: EZH1/2 dual inhibitors suppress HTLV-1-infected cell proliferation and hyperimmune response in HTLV-1-associated myelopathy
Source: Front Microbiol. 2023 Jun 12;14:1175762. doi: 10.3389/fmicb.2023.1175762 (PMC10291084; doi:10.3389/fmicb.2023.1175762)

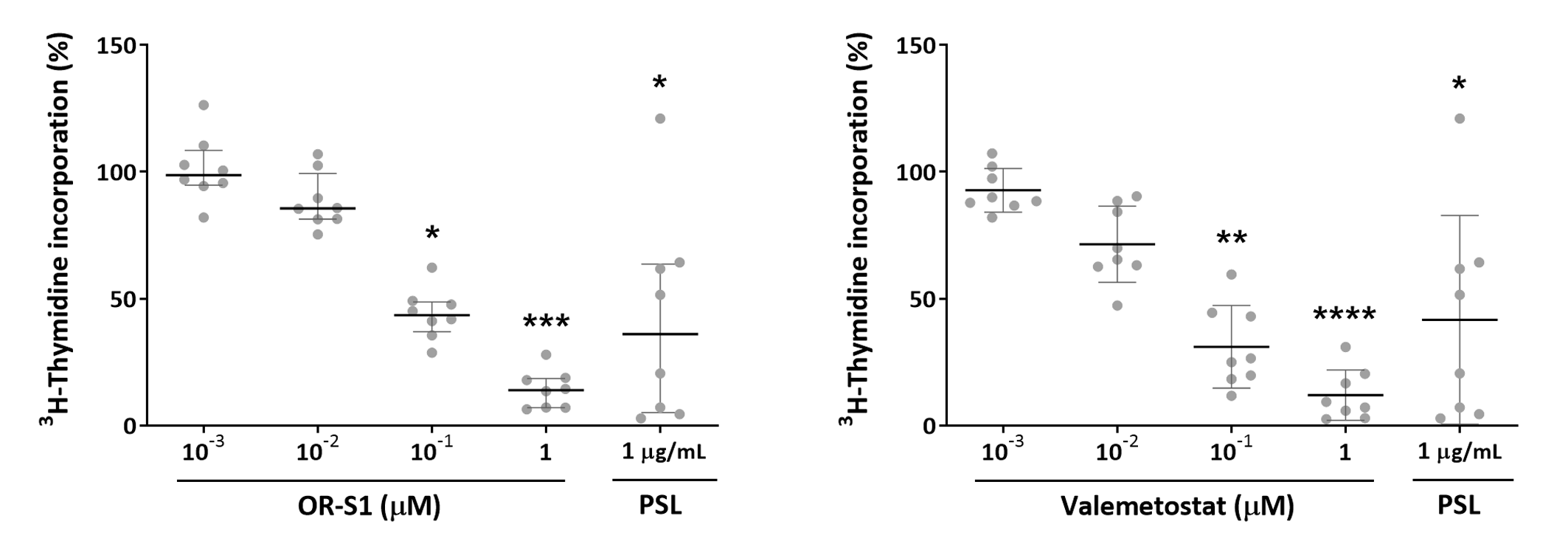

Supplement: Supplementary file 1 [file Data_Sheet_1.zip › Supplementary Figure S1.TIF]

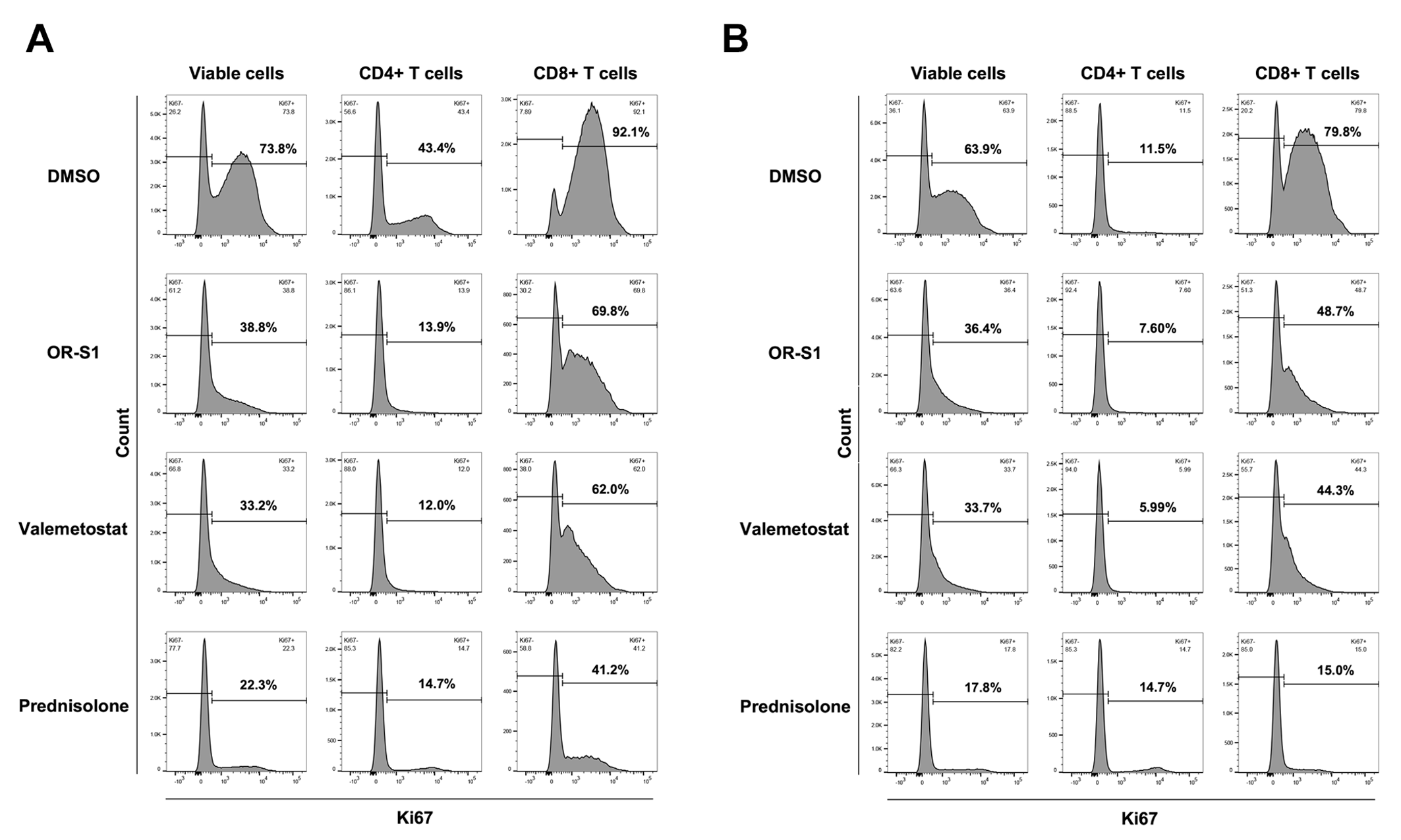

Supplement: Supplementary file 1 [file Data_Sheet_1.zip › Supplementary Figure S2.TIF]
